# Patterns of Detecting Substances of Abuse in a Large Series of Patients in Spain During 2021–2025

**DOI:** 10.3390/clinpract16090173

**Published:** 2026-09-21

**Authors:** Carmen Lara-Apolinario, Cesar Martinez-Carral, Desiree Fischetti, Adexe J. Fulgencio-González, Casimira Domínguez, Pedro C. Lara

**Affiliations:** 1 Preventive Medicine and Public Health Department, Infanta Elena University Hospital, 28342 Madrid, Spain; cala.edu@quironsalud.es; 2 Medicine Department, Fernando Pessoa Canarias University, 35450 Guía, Spain; cesarantonio.martinezcarral@osakidetza.eus; 3 Preventive Medicine and Public Health Department, Cruces University Hospital, 48903 Barakaldo, Spain; 4 Laboratory Medicine Department, Dr. Negrín University Hospital, 35010 Las Palmas de Gran Canaria, Spain; 5 Canarian Comprehensive Cancer Center, San Roque University Hospital, 35001 Las Palmas de Gran Canaria, Spain; 6 Canarian Institute of Cancer Research, 38320 La Laguna, Spain

**Keywords:** immunoassay, substance abuse, cocaine, cannabis, opioids, amphetamine

## Abstract

Aim: This study was conducted to assess, for the first time, patterns in common drugs of abuse detected in urine samples from a large series of patients treated at a tertiary university hospital and the role of age and sex in these patterns. Methods: This study included samples from all patients analyzed at the Dr. Negrín University Hospital Laboratory from January 2021 to December 2025 with positive immunoassay test results for at least one of the following substances of abuse: cannabis, cocaine, amphetamine/methamphetamine, or opioids. The samples were anonymized, and only the patient’s sex and date of birth were included in the analysis. Results: In total, there were 5019 positive results for at least one of the substances screened (cannabis, opioids, amphetamine, or cocaine). Women represented only 28.2% of all patients with a positive test (*p* < 0.0001). Substance positivity was strongly influenced by age. Cannabis positivity was observed in 2989 cases (59.9%) and was very high in the younger age groups, consistently declining towards age 65 (94% for <18 years vs. 16.2% for >65 years) (*p* < 0.0001). In contrast, amphetamine (15.9%) and opioid (13.9%) use rose consistently from 7.5% and 0.7%, respectively, among patients aged <18 years, to 61.3% and 23.1%, respectively, in the >65 age group (*p* < 0.0001). Cocaine positivity (45.6%) was very low at early ages (5.4% for <18 years group), increased in middle age (62%: 36–55 years group), and decreased in older age (11.9% for >65 years group) (*p* < 0.0001). Conclusions: With more than five thousand samples analyzed, our study sheds light on the profile of substance abuse in a large population of patients attending a university hospital. Our results can help to better adjust preventive political actions and medical care, in the context of substance abuse.

## 1. Introduction

The European Union Drug Agency’s 2026 report accounted for a total of 34.3 million adults (15–64 years) who had used cannabis, cocaine, or amphetamine/MDMA during the last year. In total, 133.5 million individuals (46.7% of the European population) reported lifetime use of these substances. High-risk opioid users accounted for 855,000 cases, and opioids were the principal drug in about 21% of all referrals for drug treatment in the European Union [1]. Knowledge of the extent and seriousness of the problem is essential for establishing useful health policies related to the prevention, diagnosis, and treatment of addiction to substances of abuse, as well as to the organization of protocols and care channels that facilitate the diagnosis and adequate treatment of complications derived from addiction [2].

The extent of the problem is therefore the basis on which subsequent preventive or assistance actions must be based. The Spanish Observatory of Drugs and Addictions (OEDA) systematically collects information from multiple sources and data systems on drug use, its consequences, and interventions aimed at both demand reduction and supply control [3]. The 2025 edition presents detailed results of the Survey on Alcohol and Drugs in Spain (EDADES) 2024, as well as an analysis of the main indicators relating to treatment admissions, hospital emergencies, and mortality associated with drug use, with updates on trends observed from 2023 [3]. In addition, reports from non-governmental organizations (NGOs) can provide complementary information [4,5] and are sometimes particularly relevant due to the local or regional level of the study [6].

Hospital emergency service use data [7] have been frequently used to estimate which drugs are consumed most. In Spain, some studies report data on the use of urgent care services due to drug use, most of which are based on the analysis of individual hospitals or specific subtypes of patients [7]. In other cases, data on poisonings caused by drugs of abuse are included in the analysis of all poisonings treated in the emergency department [8]. However, specific studies with large samples of patients are scarce.

The Euro-DEN Register was created in 2013 as a result of a European Commission (JUST/2012/DPIP/AG/3591) grant by researchers from 16 hospitals in 10 European countries, including the Hospital Universitari Son Espases in Palma de Mallorca and the Hospital Clinic in Barcelona, Spain [9]. Today, more European centers are participating (Euro-DEN Plus), and the survey results were updated recently [10].

The data available are usually from self-reports of consumption by patients or proxies, but the analytic values of the substances of abuse are not frequently determined [9]. The existing data show a poor correlation of self-reported substance use with analytic results [11,12]. The Canary Islands is a Spanish autonomous community that faces the problem of substance abuse [13]. It is therefore essential to reliably know data on age and sex profiles most related to the consumption of the common substances of abuse such as cannabis, opioids, cocaine and amphetamine.

This epidemiological study was conducted to assess patterns of abuse of common drugs detected by immunoassay in urine samples from a large series of patients admitted to a tertiary university hospital and to assess the role of age and sex in possible differences in these patterns of substance abuse.

## 2. Methods

This study included patients referred from emergency departments in the northern area of Gran Canaria for symptoms or signs indicating drug use and positive immunoassay urine test results for at least one of the following substances of abuse: cannabis, cocaine, amphetamine, or opioids. Samples collected from January 2021 to December 2025 were analyzed at the Doctor Negrín University Hospital Laboratory from January 2021 to December 2025. Patients with acute poisoning exclusively due to ethanol were excluded.

Samples were processed anonymously. Only data on the patients’ sex and date of birth were registered. As a result, no information was available on possible comorbidities or abuse of substances other than those included in the present study. No data were available for treatment for substance-related symptoms at admission.

Detection of drugs of abuse in urine was performed with an automated Alinity C analyzer, using homogeneous immunoassays with the manufacturer’s commercial reagents (Abbott Laboratories, Abbott Park, IL, USA). The toxicological panel included amphetamines/methamphetamines, cocaine (benzoylecgonine metabolite), cannabinoids (THC-COOH), and opiates. The results were reported qualitatively (positive/negative). A negative result indicated only that the analyte was not present in the sample or that it was present at a concentration below the kit’s threshold. A positive result indicated that the analyte was present in the sample at a concentration above the kit’s threshold.

In urine drug screening immunoassays, the Abbott kit measures analytical and clinical sensitivity and specificity (diagnostic yield) in terms of concordance with a reference technique (such as gas chromatography/mass spectrometry (GC–MS) or LC–MS/MS).

The Abbott Alinity C Cannabinoids Reagent Kit, ref. 09P5420, was used to detect cannabis. The target analyte was 11-nor-Δ^9^-THC-9-carboxylic acid (THC-COOH), and the threshold value for detection was 50 ng/mL. Sensitivity and specificity (concordance) showed a total agreement greater than 98% with the reference chromatographic method (GC–MS). Cross-reactivity with cannabidiol (CBD) is negligible (less than 0.1%), so the presence of CBD does not produce a false-positive result in the assay unless the product consumed contains undeclared traces of Δ^9^-THC. Minor cannabinoids (e.g., Δ^8^-THC, cannabinol [CBN], and cannabigerol [CBG]) can exhibit variable degrees of cross-reactivity, occasionally leading to positive screening results. The manufacturer does not report clinically relevant cross-reactivity with the synthetic cannabinoids evaluated. Therefore, this assay should not be considered a screening assay for synthetic cannabinoids. False negatives can appear for adulterated or excessively diluted samples. False-positive results can also appear if the THC-COOH metabolite adheres to the walls of plastic containers in unrefrigerated samples.

The Abbott Alinity C Cocaine Reagent Kit, ref. 09P5520, was used to detect cocaine. The target analyte was benzoylecgonine, and the threshold for detecting cocaine and its metabolites was 300 ng/mL. Sensitivity and specificity (concordance) were very high, showing analytical agreement with GC–MS of greater than 99%. The test has a very high specificity without significant cross-reactivity with common local anesthetics (lidocaine, benzocaine, and procaine). False negatives can occur in the case of degradation or hydrolysis of benzoylecgonine if the sample is stored at an alkaline pH or room temperature for prolonged periods (2).

The Abbott Alinity C Amphetamine/Methamphetamine Reagent Kit, ref. LN 09P4520, was used to detect amphetamine/methamphetamine. The target analytes/metabolites were d-Methamphetamine/d-Amphetamine. Amphetamine and its derivatives were considered present at over 500 ng/mL. For sensitivity and specificity (concordance), the technical data sheet reports the analytical performance in terms of clinical agreement with GC–MS/LC–MS; in this case, reported positive agreement was greater than 97% and negative agreement was greater than 98% at the cut-off point. The assay exhibits cross-reactivity with MDMA and MDA; therefore, these compounds may produce a positive amphetamine/methamphetamine immunoassay result when present at sufficiently high concentrations. However, the assay is not specific for MDMA or MDA, and positive results cannot be used to identify these compounds individually. Regarding new psychoactive substances (NPSs), the manufacturer does not provide validation or cross-reactivity data for novel psychoactive substances (NPSs). Accordingly, the assay should not be considered a validated screening method for NPSs, and a negative result cannot exclude their presence. Interferences and false positives can occur due to the presence of structurally related sympathomimetic drugs and amines as bupropion, labetalol, ranitidine, trazodone, phentermine, pseudoephedrine, ephedrine, phenylpropanolamine, L-methamphetamine, promethazine, chlorpromazine, isoxsuprine, and mephentermine. False negatives can occur in cases of highly diluted samples, extreme urinary pH values, or the presence of synthetic amphetamine derivatives that do not react with the antibody.

The Abbott Alinity C Opiates Reagent Kit, ref. 09P6520, was used to detect opioids. The target analyte was morphine, and the threshold for detection was 300 ng/mL. Sensitivity and specificity (concordance) were greater than 98% with respect to GC–MS/LC–MS reference methods. The immunoassay uses morphine as a calibration and reference target analyte. However, due to the high cross-reactivity of the antibody with morphine’s core structure, the assay effectively detects natural opioids and metabolites with similar molecular structures. Therefore, the substances detected included the following: (a) morphine, direct detection as the main analyte; (b) codeine, detected by high direct cross-reactivity with the antibody; and (c) heroin, detected through its main urinary metabolites, morphine and 6-monoacetylmorphine (6-MAM), which react in the analysis to yield a positive result, and the structurally related semi-synthetic hydromorphone and hydrocodone (which are detected at slightly higher concentrations according to the manufacturer’s specificity table). Complex synthetic and semi-synthetic opioids such as fentanyl, Buprenorphine, Methadone (an EDDP metabolite), oxycodone, Oxymorphone, and tramadol are not cross-reactive with the anti-morphine antibody at therapeutic or usual concentrations, so they are not reliably detected by this kit and therefore require dedicated assays. Cross-reactivity and false positive detection are possible for other susbtances, such as diphenhydramine, naloxone, and naltrexone, when present at elevated concentrations.

No confirmation using a specific analytical method such as GC–MS or LC–MS/MS was performed. Our positive immunoassay results should therefore be considered presumptive positive results rather than confirmation of substance exposure.

### 2.1. Ethics

This study was conducted in accordance with the principles of the Declaration of Helsinki and approved by the Ethics Committee of Fernando Pessoa University on 24 June 2024 (EC code: PI 005-2024) and the Ethics Committee of the Doctor Negrín University Hospital on 26 February 2026 (EC code: 2026-082-1). Given that the analysis was retrospective and the samples were pre-collected and analyzed anonymously, the ethics committees waived the need for individual consent. Consequently, written informed consent to participate was not obtained from the patients.

### 2.2. Statistical Analysis

Comparisons of rates of substance positivity between age and sex groups were assessed using the Chi-square test. A probability value of 0.05 was considered statistically significant. Statistical analyses were performed using SPSS version 26 (IBM Corp., Armonk, NY, USA) [14].

## 3. Results

A total of 5019 samples tested by immunoassay were positive for at least one of the substances screened (cannabis, opioids, amphetamine, or cocaine). Age and sex distributions are shown in Table 1. Women represented only 28.2% of all patients with a positive result for the substances analyzed (*p* < 0.0001). The 36–55 age group had the highest number of positive tests (*p* < 0.0001).

Urine sample positivity was strongly influenced by age. Cannabis positivity was observed in 2989 cases (59.9%), and positivity was very high in younger age groups, consistently declining towards 65 years of age. In fact, 94% of the samples from patients younger than 18 were positive for cannabis compared to 16.5% of the samples from those older than 65 years (*p* < 0.0001).

In contrast, use of amphetamine, representing 15.9% of the substances detected, and opioids, which were detected in 13.9% of the samples, rose consistently from 7.5% and 0.7%, respectively, in patients younger than 18, to 61.3% and 23.1%, respectively, in patients older than 65 (*p* < 0.0001).

**Figure 1:**
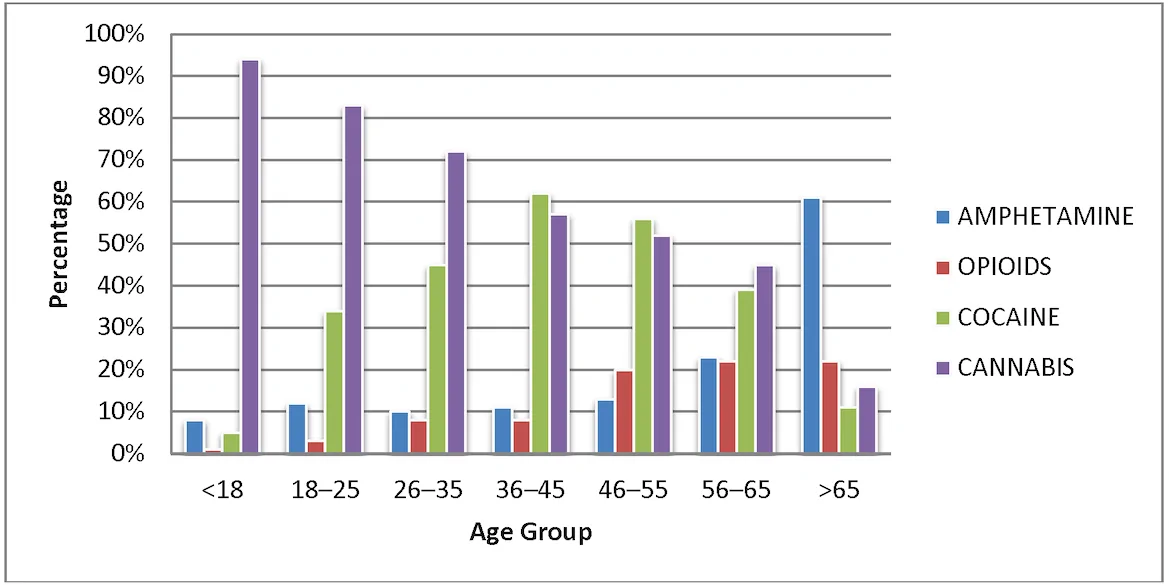
Distribution of positive urine test results for cannabis, cocaine, amphetamine, and opioids according to age.

Cocaine was detected in 45.6% of the cases. Urine sample positivity for cocaine was very low (5.4%) at early ages (patients younger than 18), increased (62%) in middle age (36–55 years), and decreased (11.9%) in patients over 65 (*p* < 0.0001) (Figure 1).

**Figure 2:**
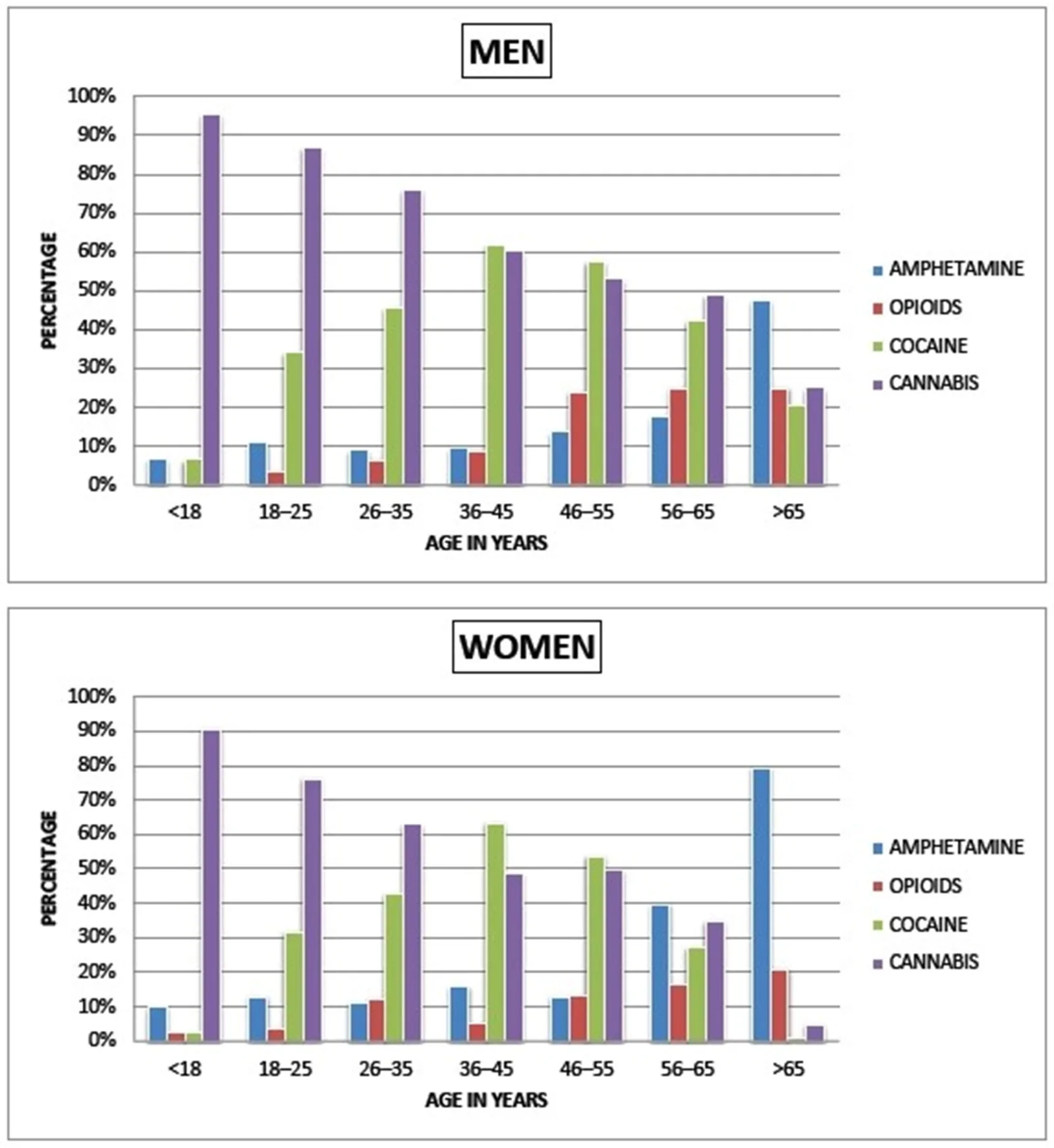
Distribution of positive urine test results for cannabis, cocaine, amphetamine, and opioids according to sex and age.

There was also a significant difference in patterns of urine sample substance positivity among men and women in the different age groups (Figure 2; Supplementary Table S1). Women had lower percentages (51.6%) of cannabis positivity than men (63.2%). This difference strongly increased in the older age group (4.8% in women vs. 25% in men) (*p* < 0.0001). Cocaine-positive test result rates were very similar between women and men in most age groups, including the 36–45 age group. Cocaine detection strongly declined in women in the older age groups. In the 46–55 age group, 53.5% of women and 57.4% of men were positive for cocaine. These differences increased in the 56–65 age group, where women had a 27.1% detection rate compared to 42.1% for men. In patients older than 65, only 0.8% of women tested positive for cocaine, compared to 20.5% of men.

Amphetamine/methamphetamine-positive test results were more common in women (22.2%) than in men (13.5%), and this difference was maintained in most age groups. The largest difference was observed in patients older than 65 (79% in women vs. 47.5% in men) (*p* < 0.0001).

Opioid positivity was slightly higher in men than in women (13.8% in men vs. 10.4% in women) (*p* < 0.0001), with a predominance of use by men aged over 36.

Concomitant detection of more than one substance was also recorded. A total of 1927 out of 5019 cases (38.39%) were positive for more than one drug in the immunoassay. The most common concomitant substances were cannabis and cocaine in 769 cases, followed by cocaine and opioids in 152 cases. The relations to age and sex were similar to those observed for single-substance detections.

## 4. Discussion

Our study reports the results of an analysis of a large cohort of patients admitted to a Spanish university hospital over the last five years, with more than five thousand assays performed. In fact, this is the largest series of drug detections in emergency departments in Europe [15]. Our study provides a comprehensive overview of the most common drugs detected in urine samples and of the sex and age variations observed.

It is important to emphasize that up until now, most available data regarding substance abuse came from surveys and self-reports by the affected persons or proxies. In most cases of acute substance toxicity, patients presenting at the emergency unit are managed based on self-reported substance use and clinical presentation [10]. We considered that relatively rapid analytical tests using immunoassays could provide relevant information, especially if no other information was available from patients’ self-reports.

The Euro-DEN Plus study [11] aimed to compare self-reported and analytically confirmed substance use in cases of acute recreational drug toxicity. They included 831 patients with available data on abuse substance analytics from a total group of 10,956 cases of acute recreational drug toxicity. The best agreement (self-reported vs. analytical determination of substances) was observed for heroin (86%) and cocaine (74%). In our opinion, this study demonstrates (a) the current lack of analytical detections of substances of abuse in patients admitted due to symptoms of substance abuse and (b) the lack of a close correlation between self-reported use and the analytical data.

The five thousand and nineteen urine samples tested for cannabis, amphetamine, cocaine, and opioids in our study demonstrated a very high level of cannabis use among younger patients, which uniformly declined toward older ages. Cocaine was prevalent in the middle-aged groups, and opioid and amphetamine use increased with age, predominating in older age groups.

Data on self-reported substance abuse from the Spanish hospitals included in Euro-DEN Plus [16] were reported for 1186 patients presenting at the emergency department. They have similar trends of substance abuse, with cocaine and cannabis detected most frequently, followed by amphetamine and opioids. Updated data on emergency consultations demonstrated that cocaine (47.8%), cannabis (44.4%), amphetamine derivatives (25.5%), benzodiazepines (8.8%), and opioids (7.3%) were the most frequently declared substances of abuse [17].

Alternative information about the use of substances of abuse can be found in analyses of city wastewater, from which the community consumption of substances of abuse can be estimated [18,19]. Data from Spain [20] align with global and European results. It is important to emphasize that amphetamine consumption was the highest in Bilbao and Barcelona and appeared extremely high compared to the rest of cities included in the study [20]. Data from the wastewater of Spanish prisons confirmed the primacy of cannabis, followed by cocaine, in the patterns of consumption [21]. Indirect consumption data were obtained from roadside tests of drivers. Cocaine (48.59%), delta-9-tetrahydrocannabinol (THC) (68.19%), opioids (20.78%), and amphetamine-like substances (16.76%) were the most common substances detected in Spanish drivers [22,23].

Our results seem to agree with the available data regarding the detection of substances of abuse [16,17,18,19,20,21,22,23,24]. Amphetamine was detected in 15.9% of the samples, which is a lower rate than in the Euro-DEN registries [16,17]. Even so, amphetamine was detected at a higher rate in our series of older women in Las Palmas, which may be related to limitations of the amphetamine/metamphetamine immunoassay used. As described above, false positives can occur due to structurally related sympathomimetic drugs and amines such as bupropion, labetalol, ranitidine, trazodone, phentermine, pseudoephedrine, ephedrine, phenylpropanolamine, L-methamphetamine, promethazine, chlorpromazine, isoxsuprine, and mephentermine.

Women represented roughly 28% of the positive tests for the substances studied in our large cohort of patients. Data from 2018 from hospitals in Spain [16] showed that 22% of women visited an emergency department due to substance abuse, while more recent results from the REDUrHE [17] showed a rate of 24.5%, and data from Spanish ONGs varied from 21.3% [4] in a nationwide survey to 27.7% [6] in the Canarian ONG survey. The Canarian ONG data agree perfectly with our own data. This allows us to conclude that the women/men ratio is consistent with the data obtained in our social environment. In our opinion, this fact is very important because further research on biological predictive factors (systemic inflammation, markers of cardiovascular risk, etc.) will be possible due to the high number of women included in our study [25]. Furthermore, stratification by age and sex of substances detected in urine samples can improve the management of symptoms related to substance abuse in hospitals. It can also provide a more accurate overview of substance abuse in a community.

Concomitant substance detection was observed in 38.39% of our cases, ranging between the 25% detected in random drivers [26] to the 47% detected in Spain [27]. Results from the Euro-DEN Plus program indicate a 37.9% rate of concomitant use of drugs apart from ethanol [15]. Concomitant substance abuse is increasing worldwide [28].

## 5. Limitations

This study has several limitations. No clinical data related to admission to the emergency department or quantitative data regarding the amount of the substances studied were available. Only cannabis, cocaine, amphetamine/methamphetamine, and opioids were analyzed. Further, the immunoassay used has relevant limitations, as described above, for each of these substances and can yield both false positives [29] and false negatives [30].

In our immunoassay, minor cannabinoids (e.g., Δ^8^-THC, cannabinol [CBN], and cannabigerol [CBG]) can exhibit variable degrees of cross-reactivity, occasionally leading to positive screening results. No clinically relevant cross-reactivity with the synthetic cannabinoids was observed [31].

A positive opiate immunoassay result should be interpreted as evidence of the presence of a morphine-related immunoreactive compound rather than as definitive evidence of heroin or other illicit opioid use. Because the assay cannot distinguish between illicit opioid exposure and therapeutic use of morphine or codeine, medication history should be considered when interpreting positive results. On the other hand, the opiate assay shows no interference or false positives for common medications for cancer pain (such as fentanyl, tramadol, or oxycodone) because their synthetic structures are not detected by the test [32].

The amphetamine/methamphetamine assay exhibits cross-reactivity with MDMA and MDA. However, the assay is not specific for either, and positive results cannot be used to identify these compounds individually. Due to their similar structures, interferences and false positives can occur for related sympathomimetic drugs and amines used to treat several common disorders [32].

## 6. Conclusions

In this epidemiological study, we analyzed rates of positivity for cannabis, cocaine, amphetamine/methamphetamine, and opioids detected by immunoassay in the largest population studied up until now, including more than five thousand samples from patients attending a university hospital. Cannabis and cocaine were the most frequently detected substances. Opioid and amphetamine use increased with age. Rates of amphetamine detection in older women were higher than expected. Our study demonstrates that the patterns of substance of abuse, estimated from immunoassay results, in a large series of patients in Spain from 2021 to 2025 were similar to those observed from self-reported data in European surveys. If confirmed by specific analytical methods, such as GC–MS or LC–MS/MS, these results would be useful for defining patterns of substance abuse in a city or region. These community-based data could help to better adjust preventive political actions and medical care in the context of substance abuse. More studies including new psychoactive substances (NPSs) are needed to improve our knowledge of substance abuse in our community.

## Figures and Tables

**Table 1:** Distribution of substance-positive tests by age and sex.

Age (years)	Women	Men	Total
<18	42 (28.0%)	108 (72.0%)	150 (2.98%)
18–25	230 (31.3%)	505 (68.7%)	735 (14.64%)
26–35	241 (25.9%)	690 (74.1%)	931 (18.54%)
36–45	332 (29.5%)	794 (70.5%)	1126 (22.43%)
46–55	286 (26.5%)	793 (73.5%)	1079 (21.49%)
56–65	156 (21.9%)	555 (78.1%)	711 (14.16%)
>65	126 (43.9%)	161 (56.1%)	287 (5.71%)
Total	1413 (28.2%)	3606 (71.8%)	5019 (100%)

## Data Availability

Data are available upon request to the corresponding authors due to ethics limitations.

## Supplementary Materials

The following supporting information can be downloaded at https://www.mdpi.com/article/10.3390/clinpract16090173/s1. Table S1: Distribution of positive test for substances by age and sex.
