# Supplementary material for: Patterns of Detecting Substances of Abuse in a Large Series of Patients in Spain During 2021–2025
Source: Clin Pract. 2026 Sep 21;16(9):173. doi: 10.3390/clinpract16090173 (PMC13605693; doi:10.3390/clinpract16090173)
Supplement: Supplementary file 1 [file clinpract-16-00173-s001.zip › clinpract-4478182-supplementary.pdf]

Supplementary Table S1. - Distribution of positive test for substances by age and sex.

	CANNABIS			COCAINE			AMPHETAMINE			OPIOIDS		
**AGE**	**WOMEN**	**MEN**	**TOTAL**	**WOMEN**	**MEN**	**TOTAL**	**WOMEN**	**MEN**	**TOTAL**	**WOMEN**	**MEN**	**TOTAL**
**<18**	38<br>90.5%	103<br>95.4%	141<br>94.0%	1<br>2.4%	7<br>6.5%	8<br>5.4%	4<br>9.8%	7<br>6.7%	11<br>7.5%	1<br>2.5%	0<br>0.0%	1<br>0.7%
**18-25**	175<br>76.1%	437<br>86.7%	612<br>83.4%	71<br>31.7%	171<br>34.1%	242<br>33.3%	29<br>13.0%	54<br>11.1%	83<br>11.7%	8<br>3.6%	16<br>3.3%	24<br>3.4%
**26-35**	151<br>62.9%	523<br>76.0%	674<br>72.6%	102<br>42.7%	313<br>45.7%	415<br>44.9%	26<br>11.3%	61<br>9.0%	87<br>9.6%	28<br>12.2%	43<br>6.4%	71<br>7.9%
**36-45**	160<br>48.5%	475<br>60.1%	635<br>56.7%	207<br>63.1%	486<br>61.6%	693<br>62.0%	51<br>15.9%	74<br>9.5%	125<br>11.4%	17<br>5.4%	68<br>8.9%	85<br>7.8%
**46-55**	141<br>49.6%	418<br>53.1%	559<br>52.2%	153<br>53.5%	451<br>57.4%	604<br>56.3%	36<br>12.9%	105<br>13.7%	141<br>13.5%	37<br>13.4%	182<br>23.7%	219<br>20.9%
**56-65**	54<br>34.8%	268<br>48.9%	322<br>45.8%	42<br>27.1%	233<br>42.1%	275<br>38.8%	61<br>39.6%	97<br>17.8%	158<br>22.6%	25<br>16.3%	136<br>24.8%	161<br>23.0%
**>65**	6<br>4.8%	40<br>2.,0%	46<br>16.2%	1<br>0.8%	33<br>20.5%	34<br>11.9%	98<br>79.0%	76<br>47.5%	174<br>61.3%	26<br>21.0%	39<br>24.8%	65<br>23.1%
**TOTAL**	725<br>51.6%	2264<br>63.2%	2989<br>59.9%	577<br>41.3%	2271<br>45.6%	2271<br>45.6%	305<br>22.2%	474<br>13.5%	779<br>15.9%	142<br>10.4%	484<br>13.8%	626<br>12.9%
